# An Embodied Approach to Understanding: Making Sense of the World Through Simulated Bodily Activity

**DOI:** 10.3389/fpsyg.2016.01914

**Published:** 2016-12-06

**Authors:** Firat Soylu

**Affiliations:** Educational Psychology Program, College of Education, The University of Alabama, TuscaloosaAL, USA

**Keywords:** biofunctional understanding, psychological understanding, embodied cognition, simulation theories, evolution of cognition

## Abstract

Even though understanding is a very widely used concept, both colloquially and in scholarly work, its definition is nebulous and it is not well-studied as a psychological construct, compared to other psychological constructs like *learning* and *memory*. Studying understanding based on third-person (e.g., behavioral, neuroimaging) data alone presents unique challenges. Understanding refers to a first-person experience of making sense of an event or a conceptual domain, and therefore requires incorporation of multiple levels of study, at the first-person (phenomenological), behavioral, and neural levels. Previously, psychological understanding was defined as a form of conscious knowing. Alternatively, biofunctional approach extends to unconscious, implicit, automatic, and intuitive aspects of cognition. Here, to bridge these two approaches an embodied and evolutionary perspective is provided to situate biofunctional understanding in theories of embodiment, and to discuss how simulation theories of cognition, which regard simulation of sensorimotor and affective states as a central tenet of cognition, can bridge the gap between biofunctional and psychological understanding.

*Understanding* is a widely used but an ill-defined concept. Colloquially it refers to meta awareness or conscious monitoring of a mental state that involves making sense of a situation or an event. It also implies a parity between one’s own mental model (e.g., of a phenomenon), and another person’s mental model (e.g., “I understand what you mean”) or an external entity that represents a model (e.g., “I understand what the text says”). Educators often use the term *understanding* as part of the goal statement of an instructional program or intervention to imply a deep form of learning, where what is understood is internalized and becomes readily available for future interpretation of events and decision making. In spite of the wide use of the term both in colloquial and academic language, *understanding* is not subject to scientific study in cognitive sciences and education to the extent that other mainstream constructs are, such as perception, learning, memory and executive function. This essay focuses on one approach to understanding, the biofunctional one, and situates biofunctional understanding in a wider embodied cognition framework, in an effort to reinterpret understanding as a bodily state (as opposed to a mere mental state), and to situate understanding as an evolutionary outcome; a response to the pressures of living in a time-pressured environment.

Biofunctionalism defines understanding as the special function of the distributed biological activity in the various subsystems of the agent’s nervous system and the immediate source of an agent’s ongoing living experience (Iran-Nejad, 2013). Understanding is not a conscious construction or acquisition of mental structures that act as models of the outside world. Rather, it is an adaptive change at the biological level as a result of situated activity. It happens even when we are not consciously paying attention to what we do. In this sense, it is like breathing (Iran-Nejad, 2013). It can be controlled and structured with conscious reflection, but it also lingers in an automatic, unconscious way. In contrast with the cognitivist characterization of understanding as acquiring permanent and abstract representations in the long-term memory, biofunctionalism defines understanding as a dynamic process where cognitive patterns elicited during understanding are transient and dynamic.

In this paper a biofunctionalist account for understanding is situated in empirical and theoretical embodied cognition research. The overall purpose is to support the following four arguments on understanding based on theories of embodiment and empirical work on bodily foundations of cognition:

(1) Understanding is biological. It is an adaptive evolutionary response to living in a time-pressured and dynamic environment.
(2) Understanding is experiential. Understanding does not follow acquisition of permanent and abstract mental representations, it is a result of situated activity.
(3) Understanding involves acquisition reactivation of transient sensorimotor and affective states.
(4) Biofunctional understanding is a prerequisite for psychological understanding (Iran-Nejad, 2013). Simulated multi-modal activity actualizes biofunctional understanding which, in turn, underlies psychological understanding. Psychological understanding makes use of perceptual symbols systems (Barsalou, 1999, 2008) to consciously access and structure biofunctional understanding.

These arguments are not separately considered here since they are all interrelated. Instead, the discussion is structured around theories of embodiment in the most salient domains of human cognition for “understanding” (i.e., social cognition and verbal skills, manual skills and tool use, and metaphorical thinking). The reflection on these three domains unfold the necessary theoretical implements to ground a biofunctional account for understanding in embodied cognition.

## Understanding

*Understanding* is a nebulous concept. Philosophers have tackled the issue of understanding and how it differs from knowing. According to some, understanding constitutes a form of knowing (Grimm, 2006), according to others (Meserve, 1981; Zagzebski, 2001; Kvanvig, 2003) understanding implies a tighter integration between the content and the subject than knowing; “when we say that we understand what others are doing or saying, we are stating something quite different than that we know. To understand is literally to stand under, to grasp, to hear, get, catch, or comprehend the meaning of something. To know is to signal that one has engaged in conscious deliberation and can demonstrate, show, or clearly prove or support a claim” (Schwandt, 1999, p. 452).

Understanding involves internalizing the content in a way that it changes our perceptions and intuitions about what is understood. For example, “when a mathematician says he understands a mathematical theory, he possesses much more knowledge than that which concerns the deductive aspects of theorems and proofs[...] He has an intuitive feeling for the subject, how it hangs together, and how it relates to other theories. He knows how not to be swamped by details, but also to reference them when he needs them” (Gregson, 2008, p. 361). This form of understanding cannot be fully explained by computational cognitive models (Penrose, 1997). From a phenomenological perspective, understanding changes the *phenomenal field* (Merleau-Ponty, 1962); the field of our experiences, how we see the things around us and how we situate ourselves. This implies a deeper form of knowing; understanding is not an isolated change in the mental representation (e.g., schema) for a given situation or knowledge domain; it represents a new state of awareness, which changes the way we perceive and respond to the world.

Approaching understanding not simply as a cerebral notion, a phenomenon that refers to a new cognitive state, but as a new bodily state that essentially changes how we respond to events in an environment requires explicating how understanding relates to action. If understanding is not merely a mental state and actually refers to acquisition of new bodily states and patterns of behavior, then how does the body support understanding? Here I respond to this question by bridging biofunctional understanding, which involve biological mechanisms that allow development of dynamic internal states to accommodate environmental changes and to allow the organism to maintain self-integrity, with psychological understanding, which is an ill-defined concept that roughly refers to having acquired knowledge structures in an abstract domain.

## Different Forms of Embodiment

Embodied cognition is not a single theory, but rather a transdisciplinary research program representing a multitude of theories on how cognition is grounded in bodily systems and body’s interaction with the environment. Kiverstein and Clark’s (2009) distinguishes between embodied approaches that regard embodiment either as a component of cognition, or as a central tenet. The perspective presented in this paper falls into the latter category. The issue of *representation* is critical to distinguishing between embodied approaches. Classical cognitivist theories (e.g., Fodor, 1983) rely on mental-representations that are symbolic, amodal, and body-independent structures in their explanations of cognition. Efforts to situate cognition in bodily activity yielded to two solutions with dealing with the problem of amodal representations; striving to provide representation-free accounts of cognition (e.g., Beer, 2000; Chemero, 2011), or defining representations as mental structures with modal content (i.e., simulations of bodily states; Barsalou, 1999, 2008). Representation-free, radical, accounts of embodied cognition face the challenge of explaining off-line and abstract aspects of cognition (i.e., representation hungry problems; see Clark, 1999). Theoretical accounts that refer to representational content as simulation of bodily states partially deal with this problem by acknowledging the need for mental structures to explain off-line and abstract cognition, and by grounding these mental representations in simulations of bodily states (Barsalou, 2008).

The two seminal works, typically cited to mark the start of the embodied cognition research program, “Metaphors We Live By” (Lakoff and Johnson, 1980) and “Autopoiesis and Cognition” (Maturana and Varela, 1980) differ both in terms of their focus, as well as the disciplines in which they are grounded. Lakoff and Johnson’s work on conceptual metaphors is rooted in a cognitive-linguistic tradition, while Maturana and Varela’s work is essentially a biological theory of cognition. The beginning of embodied cognition as a cross-disciplinary research program that encompasses not only multiple fields but also varied research methodologies yielded to distinct development of embodied theories across multiple disciplines. Today, research about how cognition is grounded in bodily processes is conducted in various fields, including evolutionary biology, cognitive neuroscience, traditional fields of psychology, robotics and artificial intelligence, and education to name a few. This expansiveness further yields to a vast and rich array of research methods (e.g., behavioral experiments, qualitative studies; neuroimaging and neuropsychological studies, cognitive modeling, linguistic analysis, phenomenological studies). This phenomenon is unique compared to previous paradigms of cognition. For example, behaviorism was characterized by conditioning experiments, and interventions developed based on these experiments in practice fields. Furthermore, people who did the original research and devised interventions based on the original research considerably overlapped, particularly early on, for behaviorism (e.g., Skinner’s work on programmed instruction and Watson’s work on advertising). Another marked difference, compared to behaviorism and cognitivisim is, how rapidly research on embodied cognition was interpreted by and influenced social sciences and the arts. Part of the reason why embodiment was appealing to social scientists was because the idea of the embodied mind, in particular its implications for a theory of mind and consciousness, were to some extent compatible with phenomenological approaches (Dreyfus, 1996).

While what I call different forms of embodied cognition originate from different traditions and make diverging claims about nature of cognition, they share some fundamental assumptions about bodily foundations of human cognition (Wilson, 2002; Ziemke, 2003). In addition, there is considerable new interdisciplinary work merging embodied theories from different orientations to provide a more unified theory of the embodied mind that encompasses different levels of explanation, such as linguistic, behavioral, neural, phenomenological (for example see Lutz and Thompson, 2003; Gallese and Lakoff, 2005).

### Simulation Theories of Cognition

Embodied approaches differ in their core claims about the role of the sensorimotor systems in cognition and their reliance on, disembodied, internal representation or cognitive structures. One, perhaps oversimplified, way to distinguish different embodied approaches would be to put them on a scale of how much they rely on internal representations, which are internal models of the external world. Even though all embodied approaches share a general notion of cognition being grounded in bodily systems, and in-context, situated activity, off-line and abstract cognition constitutes a challenge to embodied approaches. For example, while we can explain semantic content of conceptual metaphors in language based on bodily experiences, mathematical thinking, especially high-level mathematics, is hard to explain as a function of bodily activity alone (Lakoff and Nunez, 2000). Mental representations come to our help in domains where we cannot provide a fully embodied explanation.

One key notion of embodiment is the sharing of neural resources between cognitive and sensorimotor processes. According to simulation theories, cognitive processes are embodied simulations; in the sense that they make use of simulations of sensorimotor processes. Here, I use the term *simulation theories of cognition* to refer to theories positing that all cognitive processes are simulations of sensorimotor processes. Simulation theories (Barsalou, 1999; Gallese and Lakoff, 2005; Svensson et al., 2007, 2009) posit a decoupling of sensorimotor functions from their original physical inputs and outputs, in a way that these functions are redeployed during the evolutionary process to serve conceptual processing (Anderson, 2007). Embodied simulations are the source of both structural and semantic content in conceptual knowledge. Embodied simulations take place in multimodal sensorimotor networks. Unlike the conventional idea of distinct sensory and motor areas communicating through association areas, multimodality refers to the integration of sensory modalities with one another and also with motor modalities (Gallese and Lakoff, 2005). Simulation theories also provide an alternative to cognitivist notions of symbol processing (i.e., symbol crunching) by arguing that symbolic processing can be grounded in sensorimotor systems. For example, Barsalou (1999) argues that during perceptual experience association areas in the brain capture bottom-up sensorimotor patterns. Later, during the use of perceptual symbols association areas activate some of the same sensorimotor areas in a top-down manner. From this perspective the meaning of symbols (semantics) emerge from the sensorimotor simulation of relevant systems. Through experience, memories of the same components are stored in a schematic manner. The memories implement simulators of the perceptual experiences they represent. Simulators can be perceptual, proprioceptive (coding the position and movement of the body), or introspective. Abstract concepts are grounded in the combinatorial and recursive integration of simulators. Barsalou’s *perceptual symbol systems* theory (Barsalou, 1999) does not completely negate cognitive representations, but instead provides a framework for how representations (e.g., abstract symbols) can be grounded in the sensorimotor system. Not completely negating cognitive representations and symbol processing allow a higher explanatory power for grounding conceptual processing in sensorimotor systems.

In an effort to situate understanding within the framework of embodiment presented, I argue that understanding could be thought as development of bodily (sensorimotor and affective) simulations that enable the modeling of the phenomenon understood. Modeling here refers to development of a set of simulations that provides an immediate sense of the inferential structures and casual relations among the elements of the domain studied. For example, understanding arithmetic or algebra involves developing a sense for the affordances of the symbols and notations used. What makes an expert in this domain is how automatically one can make use of the perceptual representations of the mathematical symbols presented in a problem. Goldstone et al. (2010) show that across different domains of science and mathematics (including arithmetic and algebra) people rely on automatized perceptual simulations to perform high-level tasks. Reliance and further specialization of perceptual systems increase with expertise (i.e., experts rely more on these automatic perceptual mechanisms). The automatic mechanisms that develop with expertise allow the learner to provide automatic and quick task-related responses. From this perspective, understanding is about having developed the perceptual and motor mechanisms that intuitively and automatically interact with the different elements of the domain understood.

*Affordance* (Gibson, 1986) is a key concept that can bridge bodily mechanisms that help the organism to habituate in an environment, develop intuitions, make automatic predictions about future events, and seamlessly simulate opportunities for interaction based on previous interactions, and *understanding* as a psychological construct, which refers to having command of an abstract domain, having insights about how the different conceptual elements interact, and seamlessly perceive the inferential relations among them. In his original description Gibson (1986, p. 127) described affordances of an environment as “what it offers the animal, what it provides or furnishes, either for good or ill”. He also pointed out that affordances of an object are relative to an animal. The affordances change depending on the anatomy, posture, behavior, and intentions or goals of the animal. In this sense, affordance is not like inherent physical qualities, but instead an emergent theme in the interaction between the organism and the environment. Within this analogy (between physical contexts and conceptual domains), conceptual domains are like physical environments we live in. Concepts are like objects. They afford certain interactions and transformations. The affordances of concepts are relative to mental, emotional and intentional states of the cognizer. Understanding is developing a sense of affordances of the conceptual landscape; automatically activating these affordances, predicting events, and having an intuitive understanding of the inferential relations among the different elements of the landscape.

Proposing that understanding has its origins in the organism’s efforts to habituate to a physical context, and handle the pressures imposed by the environment, calls for an evolutionary account of how different bodily skills, originally evolved to respond to such environmental pressures, were eventually repurposed to serve abstract thinking and understanding in conceptual domains. Below such an account is provided across three domains of human cognition; social cognition, tool use, and metaphorical thinking.

## Evolution of Social Cognition and Verbal Skills

Action understanding is the ability to make sense of another individual’s actions by observing these actions. Early neuroscience research showed that primates have specialized systems for action understanding. This system enables the observer to mentally simulate the observed goal-directed action in an effort to make sense of the intended goal of the action, and predict the possible outcomes (di Pellegrino et al., 1992; Rizzolatti et al., 1996). Action understanding is a prerequisite for coordinated and collaborative goal-oriented group behavior. Action understanding is also a prerequisite for theory of mind skills; understanding mental states of other individuals based on their observed behaviors.

Theories of how we understand mental states of other individuals (“mind reading”) can roughly be categorized into two: According to the theory-theory (TT), mind-reading is possible by theorizing about the inner states (e.g., desires, beliefs) of another individual and predicting the observable behaviors based on the assumptions about these inner states (Carruthers, 1996). TT approaches social interaction as a disembodied, cognitive phenomenon. A second approach, simulation theory (ST), asserts that humans understand other people’s mental states by imaginatively constructing and adopting their perspective. According to ST mind-reading involves simulation of the perceived conditions of another individual, and matching the inner state of the observed individual with the resonant states of the self, i.e., states that one can understand as “perspectives I have taken” (see Gallese and Goldman, 1998 for a comparison of these two approaches).

Findings about cortical mechanisms in the monkey brain that activate perceptuomotor systems associated with the observed action during observation of goal-directed actions suggest that social skills, like imitation and mind-reading, involve simulation systems (Hauser and Wood, 2010). Studies on humans show evidence for a similar “mirroring” simulation mechanism in the human brain (Grezes et al., 2003; Mukamel et al., 2010), namely the Mirror Neuron System (MNS).

Identification of the MNS and theories of social cognition incorporating the MNS lead to embodied and evolutionary accounts for human communicative behavior (Rizzolatti and Arbib, 1998; Arbib, 2002; Gentilucci and Corballis, 2006; Fogassi and Ferrari, 2007). These accounts connect humans’ unmatched verbal skills to more ancient skills such as grasping, action understanding and imitation, to provide an integrative evolutionary explanation for language development from early primates to humans. For example, Arbib (2002, 2005, 2011) proposed that a MNS for motor behavior, especially for hand movements and facial gestures, is the antecedent of verbal communicative behavior in humans. The proposed evolutionary trajectory involves, first the development of a mirror system that matches observation and execution of hand movements for action understanding, then emergence of the ability for imitation, followed by a manual (or gesture) based communication system, and the development of the vocal system, ultimately leading to complex human languages.

## Manual Skills, Tool Use, and Social Learning

Tool use can generally be described as the use of an external physical entity to improve human manipulative capabilities. The adaptive advantage of tool use is proposed as one of the most important driving factors of hominid evolution (van Schaik et al., 1999) and learning how to use tools through imitation and social interaction co-evolved with human communicative skills (most notably verbal and theory of mind skills; Arbib, 2011). Two distinct systems are at play in regards to humans’ use and conceptual/social processing of tools.

The first system enables semantic access to the affordances of a tool merely based on sensory stimuli (i.e., without using the tool; e.g., seeing the tool). The affordance calculation system is thought to involve a special set of neurons, namely *canonical neurons*, distributed in parietal and premotor areas. In early primate studies canonical neurons were shown to charge when the monkey observes and executes grasping movements, as well as when the monkey passively observes an object that affords a grasping movement (Rizzolatti and Fadiga, 1998). Homologue canonical systems in a network of left premotor and parietal areas were found in humans when subjects were shown pictures of tools but not when they were shown pictures of animals, faces or houses (Chao and Martin, 2000; Grezes et al., 2003), which supports the idea that canonical neurons differentially respond to manipulable objects. Furthermore, Grezes et al. (2003) point out that the ventral precentral sulcus activation during observation of manipulable objects overlap with previous imaging studies involving perception of gestures, execution of hand movements or mental imagery of hand movements (e.g., Grafton et al., 1996, 1997; Chao and Martin, 2000), which suggests that identification of a tool involves activating motor circuits that are originally used during interaction with that tool.

The second system is involved in the use of a tool. Clinical case studies suggest that the two systems, for identification and use, might be dissociable (one can have disruptions in one system without affecting the other one; see Johnson-Frey, 2004 for a review). This dissociation might have allowed further specialization of the semantic system, independent of the motor component.

## Metaphorical Thinking: Mapping a Bodily Domain with a Conceptual Domain

Study of conceptual metaphors started with analysis of verbal and written language to explore how humans’ conceptual worlds are largely metaphorical. Here a metaphor is defined as a mapping from a familiar and casual source domain to a novel and possibly more abstract target domain (e.g., “I wasted a lot of time”–time as a limited resource–). Because our day-to-day experiences, intuitions, and knowledge mostly involves bodily states, corporeal experiences often constitute the source domain (Lakoff and Johnson, 1980; Johnson, 1987). In addition to mapping of a source domain with a target domain, study of metaphors involves image schemas, conceptual primitives about spatial relations (Johnson, 1987), aspect schemas, structures coding events with temporal dimension, and conceptual blends, structuring of a new domain by way of blending multiple domains (Tunner and Fauconnier, 1995).

Even though studies on use of metaphors in language provide some evidence for involvement of bodily systems in cognition, early work in this domain (e.g., Lakoff and Johnson, 1980; Tunner and Fauconnier, 1995; Fauconnier and Turner, 1998) did not focus on the biological (e.g., neural, proprioceptive, psycholinguistic) mechanisms that played a role in metaphor processing. Ideas of embodiment emerged in the early 1980s concurrently across multiple disciplines (e.g., Lakoff and Johnson, 1980; Maturana and Varela, 1980; Moravec, 1988; Brooks, 1989), though unified, cross-disciplinary theories of embodiment that bridged these disciplines (e.g., cognitive linguistics and neuroscience) emerged much later (e.g., Gallese and Lakoff, 2005). According to one such theory metaphorical thinking occurs by mental simulation of the actions defined in a metaphor (Gibbs, 2006). A mental simulation of this form would warrant activation of semantically relevant sensorimotor systems both during non-metaphorical (e.g., “grasp the apple”), as well as metaphorical (e.g., “grasp the concept”) language comprehension. A multitude of studies provide evidence for use of sensorimotor systems (which are also active during execution and observation of actions) during language processing. For example, it was shown that action-related sentences modulate relevant parts of the motor system (i.e., hands and feet; Buccino et al., 2005), handedness modulates hemispherical lateralization of premotor cortex activation during action verb understanding of manual but not non-manual action verbs (Willems et al., 2010), and listening to action verbs that involve the mouth, hand, or leg engages matching visuomotor circuits (Tettamanti et al., 2005). A ST is proposed to explain these phenomena: “The understanding of action-related sentences implies an internal simulation of the actions expressed in the sentences, mediated by the activation of the same motor representation that is involved in their execution” (Buccino et al., 2005, p. 361).

In parallel to the findings on literal processing of action verbs, Gibbs et al. (2006) reported that observing, imagining, and executing actions described in a metaphor facilitated comprehension of the metaphorical meaning. They explain these findings by arguing that (a) “People’s understanding of metaphorical language involves their engaging in embodied simulations that in the case of expressions like ‘stretch for understanding’ and ‘chew on the idea’ make these phrases both understandable and conceptually plausible” (p. 222), and (b) “Having people watch, imitate, or imagine engaging in relevant embodied actions (e.g., chewing or grasping) may enhance the degree to which they conceptualize metaphorical actions through embodied simulations” (p. 224). However, in an fMRI study Aziz-Zadeh et al. (2006) reported no neural congruence between observation of hand, foot, and mouth movements and processing of metaphorical sentences involving matching actions. Aziz-Zadeh and Damasio (2008) explained this based on a difference in processing novel and familiar metaphors. In novel metaphors the salient feature might be the non-metaphorical meaning of the action verb, where the metaphorical meaning is more salient in familiar metaphors, reducing reliance on motor processing.

## Understanding in the Wild: Bridging the Psychological and Biofunctional

So far I focused on a strand of embodied cognition that explains different forms of thinking based on simulation of sensorimotor and affective states, and reflected on this approach across three domains of cognition (i.e., social cognition and verbal abilities, manual skills and tool use, and metaphorical thinking). Going back to the discussion on *understanding*, how does understanding take place across these three domains?

Understanding is social. We often characterize our ability to make sense of others’ behaviors as “understanding”. Understanding also relates to our ability to act on the world by making, learning how to use, or using tools. And finally understanding is also used to describe the extent to which a conceptual domain (e.g., mathematics) makes sense to us, based on a metaphorical match between a new domain and one that is familiar to us. Across these three domains we “understand” through situated activity; our experiences changing both our body (e.g., muscles, structural and functional patterns in the brain) as well as our first-person experience (e.g., our awareness, the way we perceive the world).

Iran-Nejad (2013) proposes two levels of understanding; psychological and biofunctional. Psychological understanding refers to things that the understander deliberately makes sense of based on what is already consciously known, which is spontaneously provided by the biofunctional understanding. Biofunctional understanding is tacit, intuitive, and unconscious. Here I propose (1) that the biofunctional understanding is an evolutionary outcome due to selective pressures of a time-pressured environment, (2) that psychological understanding is largely based on our ability to form perceptual symbol systems (Barsalou, 1999, 2008), and (3) biofunctional understanding is a prerequisite for psychological understanding in that psychological understanding happens by perceptual symbolic structuring of biofunctional understanding.

Cognition has evolved in a time-pressured environment, which selected for mechanisms that can support production of rapid and spontaneous behaviors as responses to environmental events (Clark, 1997). There are two mechanisms that support production of such spontaneous and rapid responses; (1) Prediction and anticipation mechanisms: if the agent can simulate what might take place next based on the current events, the system can also prepare itself to produce responses before the triggering events take place (Bar, 2007; Svensson et al., 2009). (2) By automatizing the responses: through protracted interaction with the environment we develop ways of interaction that becomes automatic overtime. Automaticity takes place in direct, bodily (e.g., finger movements; Wu et al., 2004), as well as social and cognitive processing (Bargh et al., 2012).

Our perception and experience of the current moment inherently involves events that are likely to happen in the very near future. Every decision and action is based on an anticipation for what might come next given our previous experiences, current actions, and the perceptual cues in the environment. In the social context, the history of interactions, cultural norms and the observed individuals provide cues for possibilities of impending social events. We interact with non-living objects in similar ways. Affordance (Gibson, 1979) is described as the possibilities of interaction with an object based on our history of interactions. The affordance calculation system is automatic and is central to our interaction with the objects in the environment (Ellis and Tucker, 2000; Borghi, 2004; Pezzulo et al., 2010). Affordance is similar to the anticipatory or predictive systems used in social interaction. It allows for activation of necessary resources before they are needed, and therefore improves production of appropriate responses. At the phenomenological level this is experienced as a sense of what might come next and limits the possibilities of future goal-directed actions.

Understanding as a concept is a social construct that refers to a self-monitored mental state. However, a scientific study of understanding needs to ground understanding at multiple levels of analysis, such as cognitive, neural, and evolutionary. From an evolutionary perspective understanding can be grounded as a response to the pressures of living in a time-pressured environment. From this perspective what is understood becomes part of the phenomenal field and changes the responses to the environmental events. For example, understanding how to use a tool can allow spontaneous generation of responses using the tool. Similarly, understanding fractions in mathematics can allow automatic consideration of fraction relations in understanding a quantitative phenomenon. Regardless of the domain, understanding implies a change in the way we see the world, which results with changes how we respond to the events in the world we live in.

Even though understanding assumes meta-awareness and self-monitoring of mental states, which is arguably unique to humans, an evolutionary approach would require tracing the antecedents of understanding. Previous work on biology of cognition provides some operational constructs that can help building an evolutionary theory of where understanding comes from.

### Autopoiesis and Biofunctional Understanding

In “Autopoiesis and Cognition” Maturana and Varela (1980) present a theory of how physiological functioning grounds cognition. Based on his earlier studies on the vision of frogs and pigeons, Maturana proposes that considering vision, and perception in general, as the mapping of an objective, external world, was an inadequate approach. This representationalist approach could not explain a multitude of cases where the sensory experience interacts with certain features of the perceived (e.g., geometrical features interacting with color distinctions), or with the situated activity of the observer, particularly in time-pressured activity. Maturana developed a new approach, in which the activity of the nervous system is considered to be determined only by the nervous system itself. The external stimuli only have the role of triggering an internally determined activity of the nervous system. This approach has a lager implication; perception is not viewed as receiving input from an external reality but the activity of constructing a reality. Maturana and Varela (1980) characterized living things as self-referential, self-constructing and autonomous units. They described a cognitive system as a system that defines a domain of interactions for maintaining itself, and the cognitive activity as acting in this domain.

Autopoiesis (Maturana and Varela, 1980; Maturana, 2002) is a concept that was initially proposed to explain processes that help biological cells maintain self-integrity. It defines an operationally closed system; with internal processes that are dynamic and ever changing to accommodate the changes in the environment and the demands of survival. Autopoiesis represents a form of sensory-motor coupling. Unlike the information processing approach where sensory inputs are internally processed to produce motor outputs, sensory-motor coupling represents integration of perception and action. Perception is guided or modulated by motor experience and the motor behavior overlaps with perceptual experience. For example, perception and categorization of objects in the environment involves motor simulation of interaction with these objects (Ellis and Tucker, 2000). In this sense, the previous motor experiences change the way the objects in the environment are perceived. From this perspective cognition is defined as “… the capacity that a living system exhibits of operating in dynamic structural congruence with the medium in which it exists” (Maturana, 2002, p. 26).

Autopoiesis is a useful construct in explaining how the cognizing agent changes its internal structure to accommodate the demands of the environment in which it is situated. It also describes an unconscious set of processes and states that overlap with the biofunctional understanding put forth by Iran-Nejad (2013), “Biofunctional is the kind of understanding that is caught spontaneously, rather than caused deliberately, by the understander […] It is regulated effortlessly by some evolution-sculpted combination of multiple internal and external sources working together simultaneously […]The good news is that biofunctional understanding continues, even in the absence of psychological understanding just as breathing occurs in the absence of taking deep breaths or smoking and healing occurs even in the absence of nursing” (pp. 4-6). Biofunctional understanding, then, can be considered as an autopoietic phenomenon, which sets the foundation for psychological understanding. For example, as discussed before, basic competencies, like action understanding and affordance calculation, which originally evolved to support an accord between one’s own internal states and the environment later (adaptive coupling) were repurposed for more abstract forms of, oﬄine, thinking. Predicting future events in an environment and planning actions to prepare for these predicted events can be considered autopoietic functions in a more broadly defined way. The point made here is that there is no discrete difference between biofunctional understanding and psychological understanding; we can see the relation between these two as part of a spectrum, with unconscious, low-level biological processes serving autopoietic functions on one end, and oﬄine cognition systems that use embodied simulations to predict and plan for future events, and involve what we refer to as abstract thinking on the other.

### Perceptual Symbol Systems (PSS) and Psychological Understanding

As mentioned earlier, embodied theories that target a representation-free account of cognition are challenged with aspects of oﬄine cognitive processing (for example mathematics). Perceptual Symbol Systems (PSS) theory (Barsalou, 1999, 2008) meets the representation problem by bridging classical theories of cognition (e.g., symbolic computation) with embodied (grounded) accounts (e.g., embodied simulations). PSS argues for a single, multi-modal simulation system tightly coupled with the linguistic system. According to PSS cognitive processes, implicit memory, working-memory, long-term memory and conceptual knowledge, differ in terms of the mechanisms used to capture multimodal states that are simulated during processing. From the PSS perspective implicit memory matches with what is described in biofunctional understanding. Implicit memory is automatic, unconscious, pre-linguistic and produces effects like pattern completion and priming (Barsalou et al., 2003). Working memory uses a similar simulation system but maintains an active modal representation in frontal areas of the brain, only temporarily. Long-term memory keeps episodic events in the form of modal simulations in the frontal and medial temporal systems. Conceptual knowledge is comprehensive and uses a distributed network of frontal, parietal and temporal systems. Barsalou (2005) also proposes that nonhuman animals have a comparable multi-modal simulation system. Differentially though, humans’ conceptual capabilities arise from the interaction between language and simulation systems. PSS is unique among simulation theories of cognition in that it does not refuse cognitive symbol systems *per se*, but rather grounds them in modal (sensorimotor/affective simulation) systems.

Perceptual Symbol Systems bridges the biofunctional with psychological understanding, and pre-theoretical with theoretical cognizing. Based on a mixed PSS and biofunctional interpretation, while the biofunctional understanding leads to an intuitive, unconscious and pre-linguistic sense of the world, psychological understanding makes biofunctional understanding accessible and structured through use of perceptual symbols.

## Conclusion

Understanding is a widely used but a nebulous concept that does not have a clear operational definition both in its colloquial and scholarly use. This is unlike other constructs in psychology. For example, *working memory* has been widely studied experimentally, there are some multitude theories of how it works at different levels (e.g., behavioral, cognitive, neural), and there are well established notions about its different components (e.g., phonological loop). This is not the case for understanding; not because understanding refers to a psychological state humans experience less often than working memory, but more because of the difficulty of operationalizing understanding from a third-person observer’s perspective. Working memory can easily be tested using a simple task (e.g., digit-span task), there are a multitude of cognitive models that explain its function (see Baddeley, 2012), and its neural correlates have been well studied using relatively simple paradigms. Understanding is not nearly as well defined as working memory, there are no established paradigms to measure it, and it is hard to provide theoretical models for it without philosophizing about the “hard problem” of consciousness (Chalmers, 1995). One important feature of understanding that distinguishes it from other widely studied psychological constructs is that it is impossible to define it without referring to, first-person, phenomenology of the experience of understanding. It is difficult to distinguish understanding from not understanding (or lack of understanding) from a third-person perspective (i.e., based on behavioral data), without reducing understanding to a form of memory acquisition or retrieval.

The first-person experience of understanding refers to a state of harmony between the internal emotional and mental states, and the perceived, external states of the world. Here I argue that one key to understanding *understanding* is to trace back the biological mechanisms for adaptive coupling; systems evolved to maintain self-integrity in the face of environmental changes. Then our task is to consider evolution of cognition as part of an ongoing evolutionary trend to develop adaptive systems that can help maintain self-integrity of an organism. For humans this processes led to unmatched skills for social communication and coordination, building and using tools, and constructing conceptual worlds based on metaphorical relations with bodily experiences. Based on theories of embodied cognition that consider simulation of sensorimotor and affective states a central tenet, I propose that understanding is a match between the predicted states by way of embodied simulations and the external states presented by the environment. Based on the metaphor of a cognitive domain as a landscape of concepts, understanding is characterized as developing an intuitive sense of the affordances of elements in a conceptual landscape and their casual interactions.

From the evolutionary perspective presented, psychological understanding is an extension of biofunctional understanding. Understanding in a conceptual domain relies on overlapping mechanisms as biofunctional understanding. Previous research about the centrality of metaphorical thinking in human cognition provides an account of how bodily experiences enable conceptual thinking based on metaphorical relations between the bodily source domain and the target conceptual domain (Gibbs et al., 2004; Gallese and Lakoff, 2005). Similarly, mechanisms of understanding in bodily domains are not markedly different than understanding in conceptual domains.

As previously mentioned understanding is difficult to study without considering the first-person experience of understanding (Iran-Nejad et al., 2015). Future research should focus on characterizing the first-person experience of understanding across neural, cognitive, and behavioral levels. Research on insight, a concept closely related to understanding; but one characterizes a more instantaneous experience, exemplifies this approach and can constitute a model for research on understanding (Kounios and Beeman, 2014).

## Author Contributions

The author confirms being the sole contributor of this work and approved it for publication.

## Conflict of Interest Statement

The author declares that the research was conducted in the absence of any commercial or financial relationships that could be construed as a potential conflict of interest.

The reviewer WS and the handling Editor declared their shared affiliation, and the handling Editor states that the process nevertheless met the standards of a fair and objective review.
